# Fetal intracranial hemorrhage in a case of 16p microdeletion

**DOI:** 10.1515/crpm-2021-0064

**Published:** 2022-08-11

**Authors:** Margarita Álvarez-de-la-Rosa Rodríguez, Mercedes Hernández-Suárez, Ana Isabel Padilla-Pérez, Ylenia Dévora-Cabrera, Walter Plasencia Acevedo

**Affiliations:** Obstetrics and Gynecology Service, Hospital Universitario de Canarias, La Laguna, Tenerife, Canary Islands, Spain

**Keywords:** fetal intracranial hemorrhage, fetal ultrasound, genetic counseling, microdeletion 16p11.2, prenatal diagnosis

## Abstract

**Objectives:**

Intracranial hemorrhages are common events in premature infants but in fetal life those incidents are often of ominous prognosis and unknown etiology.

**Case presentation:**

We present the diagnosis, evolution and management of a fetal hemorrhagic accident associated with an inherited maternal microdeletion of the chromosome 16 short arm. Abnormal neurosonography in routine second trimester ultrasound led to follow up. Fetal germinal matrix hemorrhage along with severe asymmetric ventriculomegaly and a secondary periventricular cyst developed in the early third trimester. Array CGH showed microdeletion 16p11.2.

**Conclusions:**

This microdeletion had not been previously associated with fetal intracranial hemorrhage.

## Introduction

Ischemic or hemorrhagic accidents *in utero* are extremely rare events of ominous prognosis and no effective treatment known that can lead to neurodevelopmental impairment [1]. These accidents are most commonly diagnosed in the newborn. Besides, fetal stroke is associated with prematurity, metabolic disease, intertwin transfusion syndrome, infectious or drug exposure, trauma or coagulation disorders. In absence of these causes a genetic etiology can be suspected [2]. In a recent meta-analysis the authors found several genes related to idiopathic antenatal intracranial hemorrhage: pro-inflammatory cytokines, collagen genes and X linked GATA1 gene mutation [2]. On the other hand, fetal microdeletions usually manifest *in utero* as malformations and are suspected upon the finding of ultrasound anomalies or soft markers.

Microdeletion 16p11.2 has been related to developmental delay, intellectual disability, and/or autism spectrum disorder, obesity, and an increased frequency of congenital defects with IQ being even in the normal range [3]. The deletion, which was first reported in individuals with autism spectrum disorder and learning disabilities, has been related to a variety of neuroimaging findings such as posterior fossa malformation and periventricular heterotopia [4] but has not previously found associated with fetal intracranial hemorrhage.

We present the diagnosis, evolution and management of a fetal hemorrhagic accident associated with a maternal inherited microdeletion of the chromosome 16 short arm.

## Case presentation

We present the case of a 39 year-old healthy patient with a history of infertility and no consanguinity. The patient was overweight (BMI 28.9) and this was her second pregnancy after a previous miscarriage. This pregnancy was achieved by embryo transfer after two failed *in vitro* fertilizations.

The first trimester aneuploidy screening ultrasound at week 12 + 3 was normal with CRL 55.6 mm and nuchal translucency 1.6 mm (59 centile). The results of the first trimester combined screening were: MoM PaPP-A 1.42, MoM HCG-Beta 1.46, trisomy 21 risk 1/1544.

**Figure 1:**
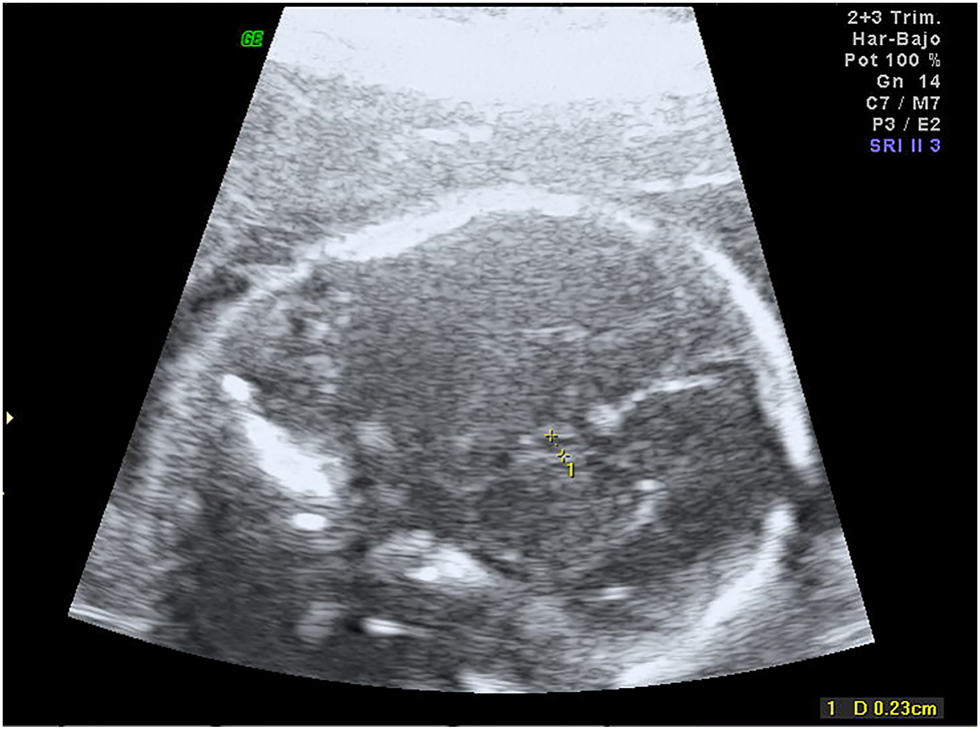
Transverse view of the fetal head at 22 weeks gestation showing small cavum septum pellucidum.

Routine ultrasound scan at 22 week gestation showed small cavum septum pellucidum (2.3 mm; −2 SD for gestational age) (Figure 1), as well as parallel anterior frontal ventricular horns. Hypoplasia of corpus callosum was suspected and reevaluation took place in a week. At 22 weeks gestation corpus callosum was 15 mm and posterior ventricular horns measurement was 8 mm. Once informed, the couple opted for invasive testing and molecular karyotype.

At 26 weeks fetal growth was within normal percentiles, corpus callosum was normal and posterior ventricular horns measured 12.8 mm. Array could not be assessed due to contamination but conventional karyotype was normal. New array and maternal serologic TORCH analyses were offered and performed.

**Figure 2:**
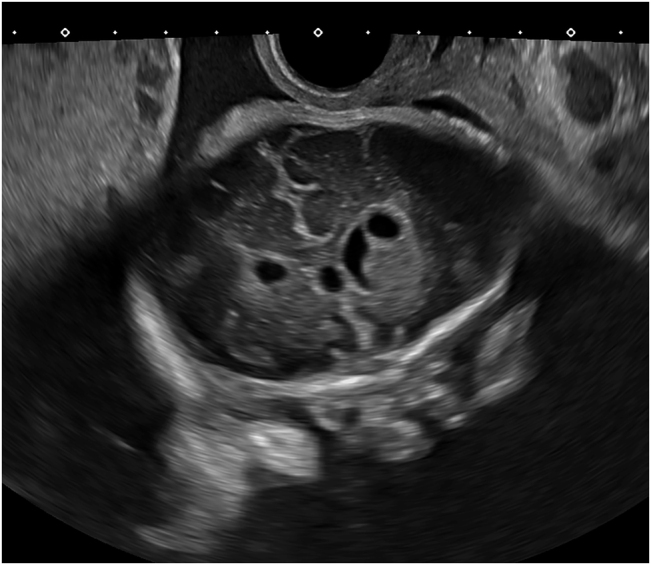
Transvaginal ultrasound at week 32 showing asymmetric ventriculomegaly and periventricular cyst.

At 30–32 weeks fetal growth remained normal and asymmetric ventriculomegaly developed, the right ventricle posterior horn reached 14 mm and there were signs of germinal matrix hemorrhage along with a secondary periventricular cyst (Figure 2, Supplemental Material, Videos 1 and 2). There was periventricular echogenicity and a complicated periventricular cyst. Medium cerebral artery Doppler showed no signs of anemia. Thrombophilia, infectious diseases screening (toxoplasmosis, parvovirus, cytomegalovirus, listeria), and antiplatelet antibodies studies were negative. CGH Array demonstrated a microdeletion in the short arm of chromosome 16, 16p11.2 (28682192_29076269)x1.

**Figure 3:**
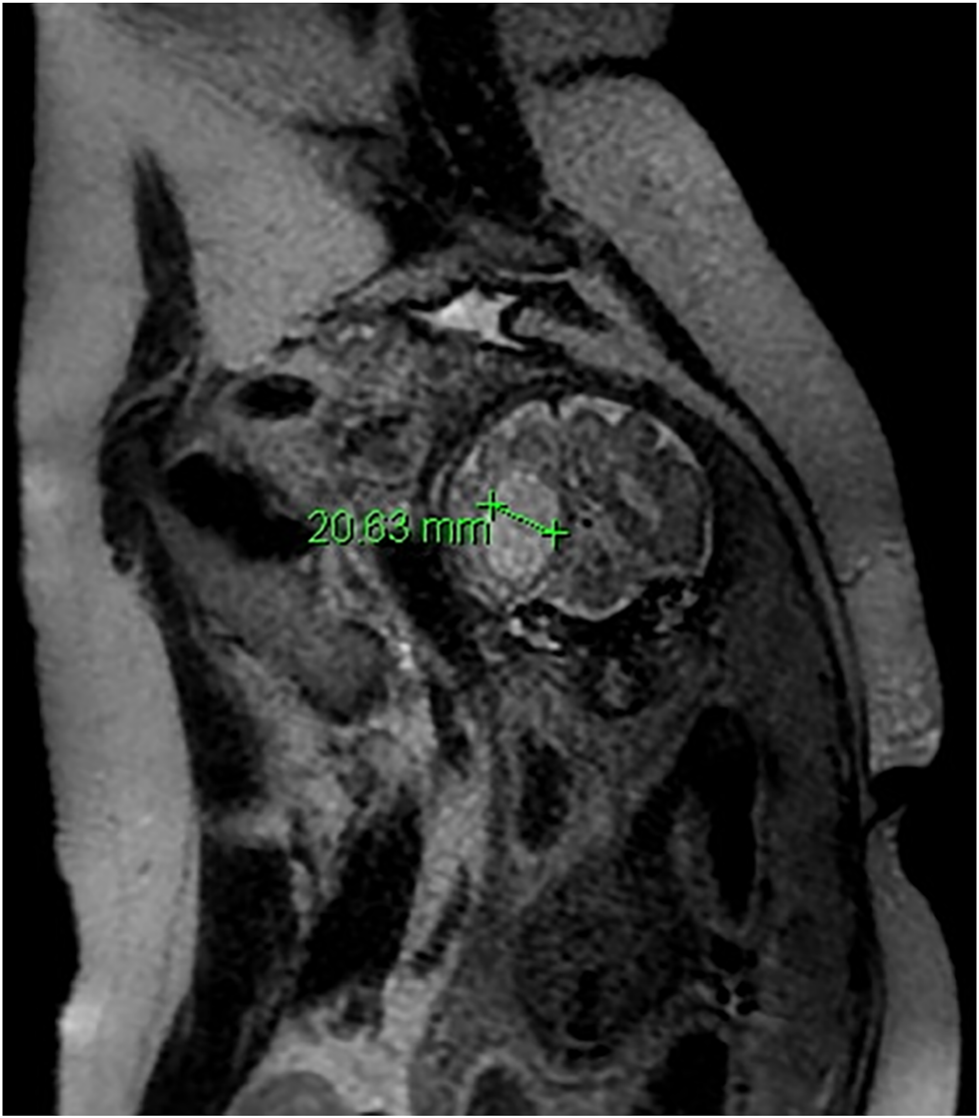
Fetal MRI at week 32 showing severe ventriculomegaly.

**Figure 4:**
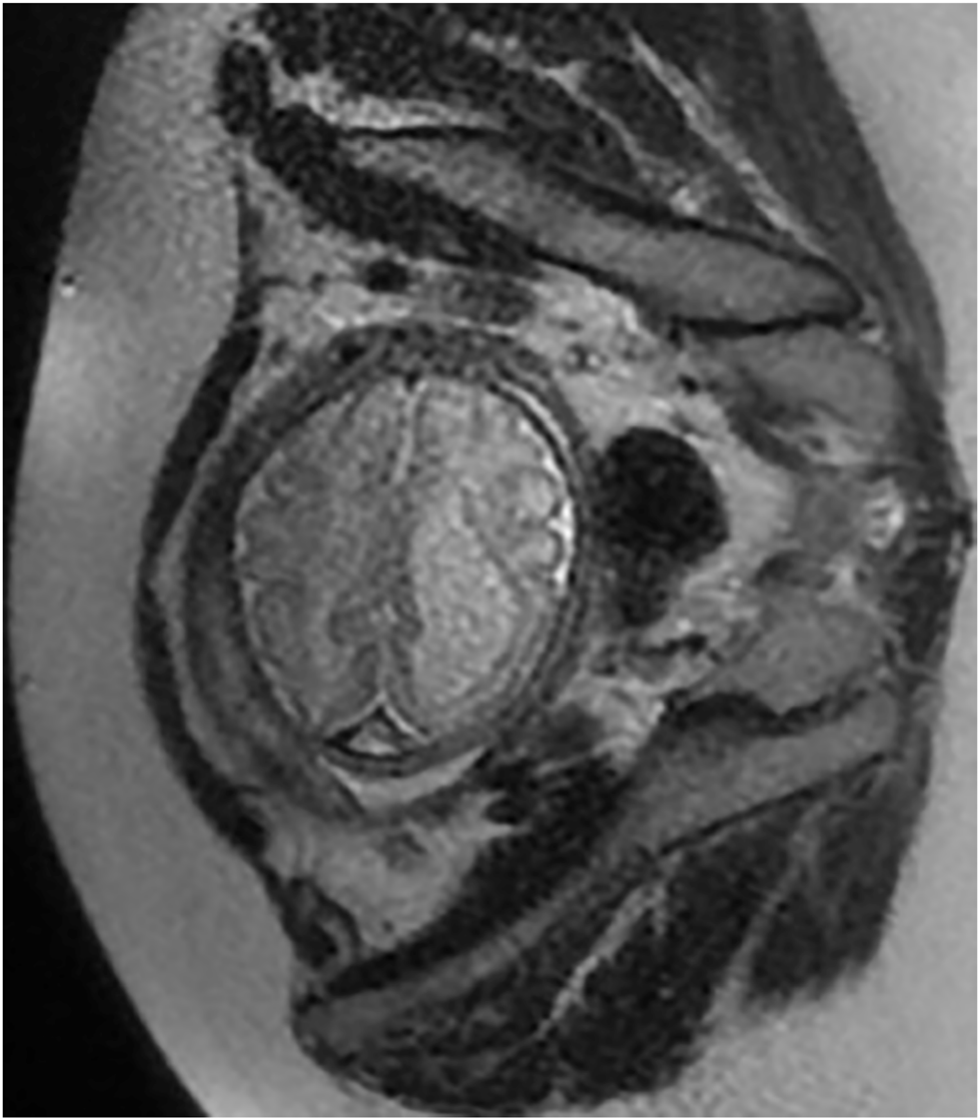
Fetal MRI at week 32 showing axial view of asymmetric ventriculomegaly.

Magnetic resonance imaging at week 32 showed severe dilatation of the left cerebral ventricle up to 20 mm and altered subcortical sign in the left hemisphere suggestive of ischemical, toxic vs. infectious insult (Figures 3 and 4).

Parents underwent counseling by a multidisciplinary team including ann a neurology pediatrician and a geneticist. The patient opted for pregnancy termination and refused necropsy. Fetal specimen showed normal phenotype. Severe postpartum hemorrhage occurred due to uterine atony and resolved after medical treatment and uterine tamponade.

Carrier status study was offered to the couple and the mother was found to be a carrier of the same deletion. This copy number variant implies a 50% risk of recurrence.

The patient opted for embryo donation and two years later gave birth to a healthy newborn after an uneventful pregnancy which ended in labor induction for prolonged pregnancy. Severe postpartum hemorrhage recurred again in absence of placental abnormalities and was controlled with conservative management.

## Discussion

Fetal cerebral hemorrhagic insult has not been previously associated with 16p11.2 microdeletion to the best of our knowledge. We could not find other causes for the fetal intracranial hemorrhage or the patient’s recurrent postpartum hemorrhage.

Fetal hemorrhage developed in the late second trimester and was diagnosed upon follow up of suspected hypoplastic corpus callosum. Cavum septum pellucidum can be seen between weeks 18–37 in the normal fetus; non visualization has been described in a variety of cerebral anomalies, especially in cases of corpus callosum hypoplasia or agenesis [5]. Normal ranges have been described by Falco et al. [5]: in week 21 cavum should measure between 3.4 and 6.2 mm. In the present case it was below −2 ds The diagnosis of short corpus callosum can be a false positive if made at an early gestational age, therefore, measurement should be repeated at least 4 weeks after the routine second trimester ultrasound.

In the present case, the 390 m kb microdeletion is associated with neurodevelopmental anomalies with a penetrance of 62.4% [6]. The deletion of the 16p11.2 region is linked to variable phenotype including obesity, behavioral disturbances and craniofacial dysmorphism [7], all of which our patient did not show. Ventriculomegaly and periventricular nodular heterotopia with seizures has been described in children affected with this deletion [4]. Chung et al. recently published a review on the medical, behavioral and neurological characteristics of deletion or duplication carriers [7]. They concluded that the deletion is most often *de novo*, but is inherited in approximately 7% of probands; it is related to autism, motor coordination difficulties, seizures, obesity, etc. and there is significant phenotypic variability among carriers. Bijlsma [8] reported on an adult who had a small deletion (200 kb) and worked as a truck driver and had learning difficulties as a child, he passed the microdeletion on to his son, who had a learning disability. Our patient works as technician in a research laboratory and has normal phenotype and no history of other bleeding disorder.

Prenatal diagnosis of the presence of this microdeletion has only been reported once in a case of fetal diaphragmatic hernia [9]. Gelfand et al. [10] studied 13 cases of perinatal arterial ischemic stroke and found that apolipoprotein E polymorphism may confer genetic susceptibility for perinatal arterial ischemic stroke. This gene is located in chromosome 19. Other authors have not found any genetic underlying condition in a series of fetal intracranial hemorrhage [11].

We hypothesize that the microdeletion was responsible for an structural abnormality that led to hemorrhage, although the lack of necropsy impairs a definite etiologic diagnosis.

We can conclude that after the diagnosis of suspected fetal intracranial hemorrhage a diagnostic workup is recommended and should include CGH array with the possibility of extending to exome.

## References

[1] Dunbar MJ, Woodward K, Leijser LM, Kirton A (2021). Antenatal diagnosis of fetal intraventricular hemorrhage: systematic review and meta-analysis. Dev Med Child Neurol.

[2] Cavaliere AF, Turrini I, Pallottini M, Vidiri A, Marchi L, Perelli F (2021). Genetic profiling of idiopathic antenatal intracranial haemorrhage: what we know?. Genes (Basel).

[3] Miller DT, Chung W, Nasir R, Shen Y, Steinman KJ, Wu BL, Adam MP, Ardinger HH, Pagon RA, Wallace SE, Bean LJH, Mirzaa G (1993). 16p11.2 recurrent microdeletion. Gene Reviews((R)).

[4] Miller D. (16p11.2). microdeletions. ..

[5] Falco P, Gabrielli S, Visentin A, Perolo A, Pilu G, Bovicelli L (2000). Transabdominal sonography of the cavum septum pellucidum in normal fetuses in the second and third trimesters of pregnancy. Ultrasound Obstet Gynecol.

[6] Rosenfeld JA, Coe BP, Eichler EE, Cuckle H, Shaffer LG (2013). Estimates of penetrance for recurrent pathogenic copy-number variations. Genet Med.

[7] Chung WK, Roberts TP, Sherr EH, Snyder LG, Spiro JE (2021). 16p11.2 deletion syndrome. Curr Opin Genet Dev.

[8] Bijlsma EK, Gijsbers AC, Schuurs-Hoeijmakers JH, van Haeringen A, Fransen van de Putte DE, Anderlid BM (2009). Extending the phenotype of recurrent rearrangements of 16p11.2: deletions in mentally retarded patients without autism and in normal individuals. Eur J Med Genet.

[9] Genesio R, Maruotti GM, Saccone G, Mormile A, Conti A, Cicatiello R (2018). Prenatally diagnosed distal 16p11.2 microdeletion with a novel association with congenital diaphragmatic hernia: a case report. Clin Case Rep.

[10] Gelfand AA, Croen LA, Torres AR, Wu YW (2013). Genetic risk factors for perinatal arterial ischemic stroke. Pediatr Neurol.

[11] Ghi T, Simonazzi G, Perolo A, Savelli L, Sandri F, Bernardi B (2003). Outcome of antenatally diagnosed intracranial hemorrhage: case series and review of the literature. Ultrasound Obstet Gynecol.

